# Molecular simulation of efficient removal of H_2_S pollutant by cyclodextrine functionalized CNTs

**DOI:** 10.1038/s41598-019-46816-2

**Published:** 2019-07-23

**Authors:** Masoud Darvish Ganji, Hadis Kiyani

**Affiliations:** 1 Department of Chemistry, Qaemshahr Branch, Islamic Azad University, Qaemshahr, Iran; 2 Department of Nanochemistry, Faculty of Pharmaceutical Chemistry, Tehran Medical Sciences, Islamic Azad University, Tehran, Iran

**Keywords:** Natural hazards, Energy, Carbon nanotubes and fullerenes

## Abstract

DFT**-**D3 calculations were carried out to investigate interaction of H_2_S and CH_4_ between numerous functionalized CNTs (f-CNTs), including hydroxyl, carboxyl, and cyclodextrin groups as potential candidates for selective adsorption and elimination of toxic pollutants. It was found that pristine CNTs as well as nanotube surface of functionalized CNTs cannot stably adsorb the H_2_S molecule (adsorption energy of −0.17 eV). However, H_2_S adsorption was significantly enhanced with different magnitudes upon the functionalization of CNT. For f-CNTs, H_2_S adsorption was accompanied by releasing energies in the range between −0.34 to −0.54 eV where the upper limit of this range belongs to the cyclodextrin**-**functionalized CNT (CD**-**CNT) as the consequence of the existence of both dispersion and electrostatic interactions between the adsorbate and substrate. Findings also demonstrated a significantly weaker interaction between CH_4_ and CD**-**CNT in comparison to the H_2_S molecule with adsorption energy of −0.14 eV. Electronic properties of the selected substrates revealed no significant changes in the inherent electronic properties of the CNTs after functionalizing and adsorbing the gas molecules. Moreover, DFTB-MD simulation demonstrated high adsorption capacity as well as CD**-**CNT ability for H_2_S molecules against the CH_4_ one under ambient condition.

## Introduction

Since one of the most important ongoing threats facing the human community is the growing concern of environmental pollution, which affects the lives of millions of people all around the world and requires immediate strategies to alleviate their hazardous effects on the civilization. Every day, tons of hazardous materials in the form of noxious greenhouse gases, as well as highly toxic fumes of industrial processes are directly discharged into the atmosphere especially in developing countries, which could have catastrophic consequences for habitability of our planet. For these reasons, monitoring and eliminating these toxic materials are of crucial importance over the years, and the challenge for their efficient removal and designing effective and novel adsorbent materials have been recently attracted by many researchers^1–4^. There is a close relationship between these issues and the emergence of nanostructures of various forms, such as nanotubes, nanowires, and hexagonal structures, which intrigued the scientists to incorporate their extraordinary properties into the fabrication of novel hybrid materials that exhibit exclusive capabilities in different fields^5–9^. Excellent physical and chemical properties of carbon nanostructures have drawn a great deal of attention to the invention of efficient materials, which show combined properties of high mechanical stability in addition to the specific tendency toward different molecules^10–12^. Chemical and electronic properties of the carbon nanostructures can be easily tailored by introducing additional chemical moieties via covalent and non-covalent functionalization, which in turn extends the scope of their application spectrum^13–15^. Several types of carbon nanostructures, such as graphene-like structures, fullerenes, and carbon nanotubes (CNTs), which are the rolled form of graphene, have been invented and possible use of these materials in the field of environmental science and engineering has been extensively explored^16–18^. In this regard, CNTs gained increasing popularity as a relatively new adsorbent for sensing and removing various organic and inorganic gaseous pollutants because of their highly porous and hollow structures and existence of strong chemical affinity between the CNTs and guest molecules. The utilization of CNTs as the gas sensor goes back to the pioneering work of Kong *et al*.^19^, which explored the electrical resistance of semiconducting single-walled carbon nanotubes upon the exposure of gaseous molecules, such as NO_2_ and NH_3._ They found out that the CNT**-**based sensors exhibit substantially higher sensitivity in comparison to the other existing solid**-**state gas sensors at room temperature^19^. Moreover, the CNTs capability as an adsorbent for hazardous materials was assessed in several experimental studies, which investigated the adsorption of a variety of small molecules, organic pollutants, and metal ions on various forms of CNTs, including closed and open**-**ended single or multi**-**walled CNTs^20–23^. According to the experimental studies, the CNTs tend to form bundle or arrays because of the existence of strong van der Waals (vdW) interactions between their surfaces^24^. This process will provide additional surface areas for physical adsorption of the guest molecules, as well as adsorption capabilities of the CNTs. The chemical modification of CNTs can also be taken into consideration for enhancing their chemical properties through direct attachment of functional groups to the terminal edges of the graphitic surface, chemical doping of impurities or the use of nanotube-bound carboxylic acids^25–27^. These different functionalization categories may introduce different chemical and electronic properties to the modified CNTs, which eventually enhances their adsorption selectivity and capacity. Consequently, a wide range of oligomeric and polymeric compounds have been used for functionalizing CNTs, which paved the way toward the development of unique CNT**-**based hybrid devices with diverse potential applications^28–31^. However, the complex nature of the experimental works, as well as the high cost and time required for conducting such experiments hinder a thorough analysis of the behavior and properties of different CNT**-**based hybrid materials, so that the needs for a precise determination of the underlying phenomena in CNT**-**based adsorbents cannot be adequately satisfied. In this regard, computational methods at quantum chemical level have been proven as an invaluable tool, which can overcome much of the constraints of experimental works and can successfully provide detailed information about fundamental aspects of physical and chemical properties of materials^32–34^. Numerous theoretical research works exist in the literature, which took advantage of computational methods and explored the potential of various materials in order to protect environment^35–40^. In a pioneering work of Zhao *et al*., adsorption of several gas molecules was investigated on single**-**walled CNT and CNT bundles^41^. It was found that the gas molecules adsorbed weakly to the SWCNTs, while the interaction was stronger with regard to the nanotube bundles. In another interesting study conducted on this topic, a combined experimental and theoretical work was done by Santucci *et al*. on CNT**-**based systems for CO and NO_2_ sensing applications^42^. The researchers observed that both gas molecules adsorbed weakly to the CNT with almost no charge transfer between the tube and molecules. Findings showed that the CNT exhibits semiconducting like temperature dependence and a *p***-**type response upon the exposure to the NO_2_, while no response was found for the CO gas. As most of the theoretical and experimental works in literature are concerned with the pristine CNT for adsorbing gas molecules, the literature lacks a detailed exploration about the possible use of functionalized CNTs (f-CNTs) as an adsorbent for toxic gases. Thus, it is of high importance to conduct a thorough analysis regarding the potential of these substrates in environmental applications. This issue has been addressed in this theoretical study and its results are hoped to accelerate developments toward designing novel and selective f-CNT nanomaterials for eliminating toxic gases. The following sections present further information regarding computational procedure and the obtained results.

## Computational Method

All**-**electron DFT calculations were carried out using the ORCA quantum chemistry software version 4.0.1 within the generalized gradient approximation (GGA) framework^43–45^. The exchange-correlation functional were selected as the revised version of Perdew-Burke-Ernzerhof functional (so-called revPBE) coupled with the third version of Grimme’s atomic pair-wise dispersion correction combined with Becke-Johnson damping, which accounts for the long-range dispersion interactions in an empirical fashion^46–48^. Selecting this computational method, which is called revPBE-D3BJ, has exhibited the best performance and accuracy in Grimme’s GMTKN30 benchmark set for studying the reaction energies and non-covalent interactions^49^. The geometries were optimized by Ahlrichs split-valence def2-SVP basis set and subjected to a basis set superposition error (BSSE) corrected single point energy calculation using the def2-TZVP basis set^50^. The coulomb part was integrated by the resolution of identity (RI) approximation utilizing the def2-TZVP/J auxiliary basis set^51,52^. All geometries were optimized to meet NormalOpt criteria in ORCA conventions, and convergence criteria for the SCF calculations were set to VeryTightSCF in order to reduce numerical noises. The adsorption energy values were calculated by the following equation as the total energy difference between the final state and initial states:

1 $${{\rm{E}}}_{{\rm{ads}}}={{\rm{E}}}_{{\rm{complex}}}-({{\rm{E}}}_{{\rm{substrate}}}+{{\rm{E}}}_{{\rm{adsorbate}}})-{{\rm{E}}}_{{\rm{BSSE}}}$$

where the incorporated terms respectively denote to total energies of the gas-nanotube complexes, substrates (individual nanotubes), and adsorbate (individual gases). *E*_BSSE_ accounts for the introduced error in energy calculations as the consequence of basis-set incompleteness, which is eliminated by counterpoise method in this study^53^.

Molecular dynamics simulation based on the density functional tight binding (DFTB**-**MD) was carried out by DFTB+ code^54^. DFTB method employs a second**-**order expansion of the Kohn–Sham total energy in DFT with respect to the charge density fluctuations. The second-order approach is equivalent to a common standard self**-**consistent charge (SCC) scheme, which is a transparent, parameter-free, and readily computable expression for the generalized Hamiltonian matrix elements. It utilizes a tabulated set of integrals derived from *ab initio* DFT calculations^55^, which leads to a considerable acceleration of the calculation procedure. Further details about the method were found elsewhere^54–57^. We used the Slater–Koster (S–K) type parameters^58^ for systems under study. In addition, the dispersion corrections for non-bonding vdW interactions, which influence stability of the systems under investigation, were considered by the Slater–Kirkwood type model^59^.

We evaluated the adsorption capability of the f-CNTs system with DFTB**-**MD simulation. The canonical regime was considered, in which thermodynamical system under consideration was described by the number of particles N, volume V, and temperature T as variables. The MD time step was set to 1.0 fs and the whole system was in contact with Andersen thermostat^60^ having temperature 300 K.

## Results and Discussion

### Interaction of H2S and CH4 with pristine CNT

**Figure 1:**
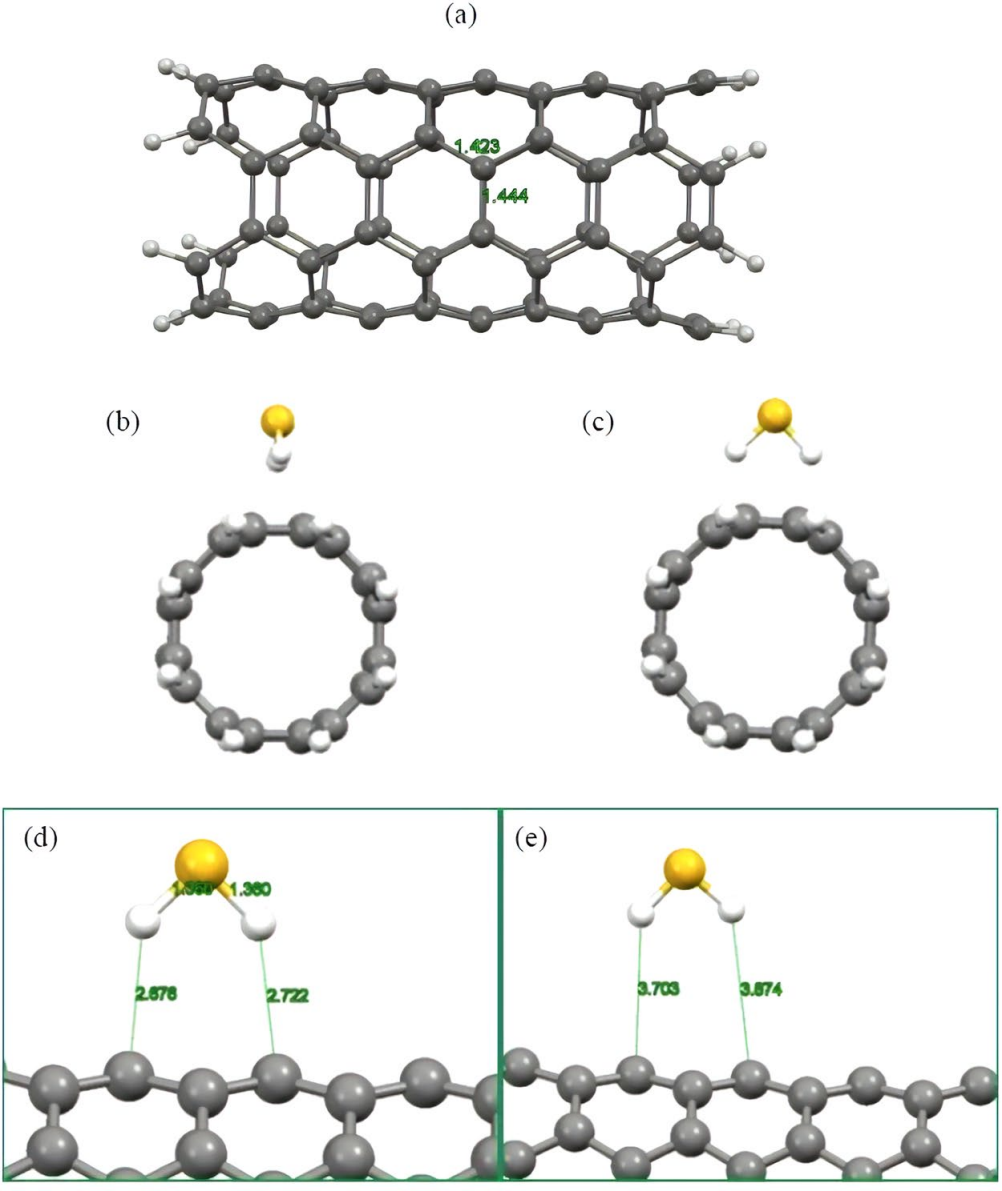
Schematic representation of (**a**) the optimized structure of CNT, (**b**) parallel and (**c**) perpendicular initial configurations of the H_2_S molecule onto the CNT. Optimized structures of the H_2_S molecule above the surface of pristine CNT (**d**) with and (**e**) without the inclusion of long-range dispersion forces. Atom color code: grey: carbon; white: hydrogen; yellow: sulfur.

**Figure 2:**
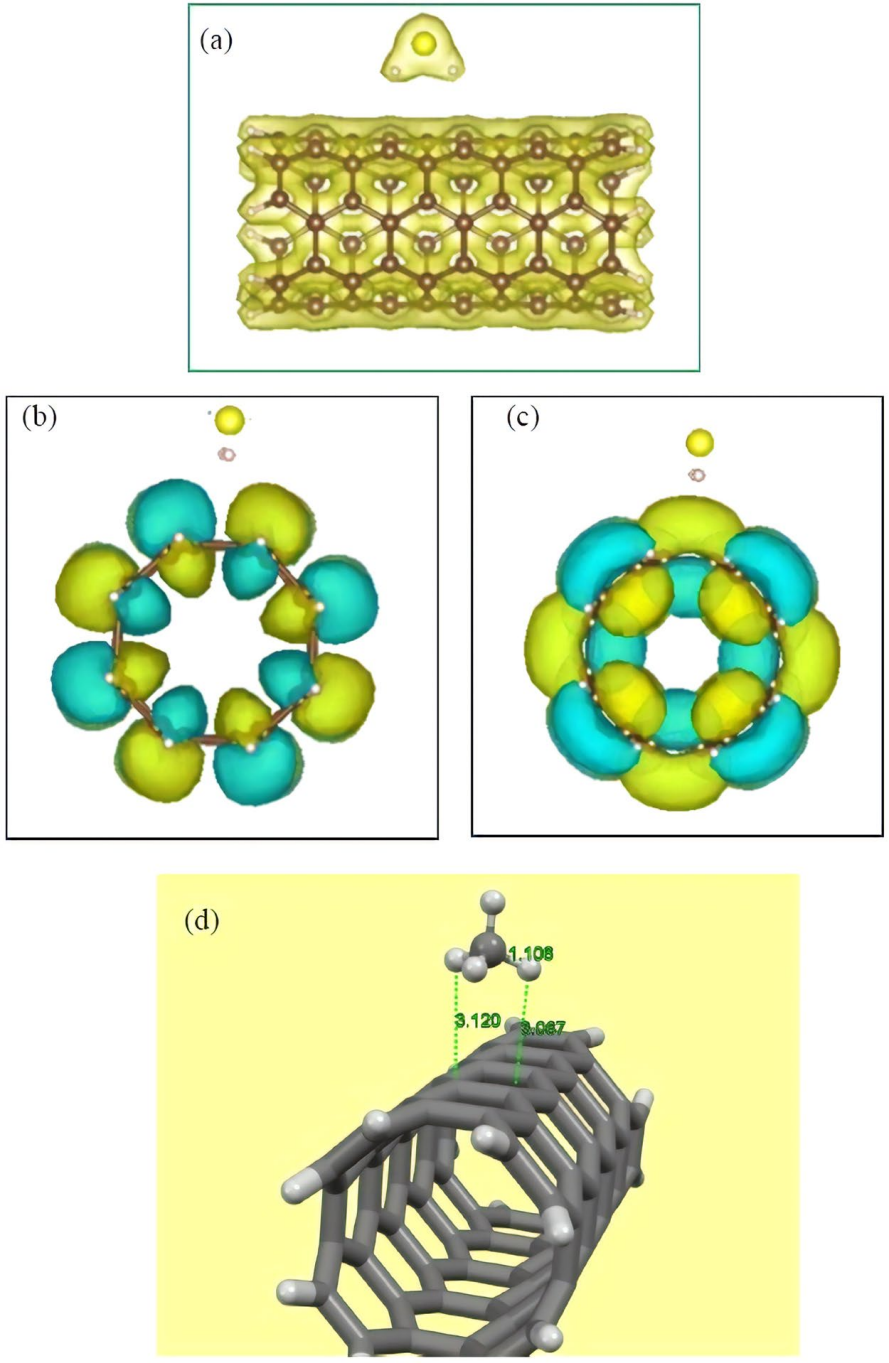
(**a**) Total electron density map of the most stable adsorption pattern for the H_2_S molecule above the CNT. (**b**) HOMO and (**c**) LUMO electron densities of the H_2_S/CNT complex. (**d**) Optimized structure of the CH_4_ molecule onto the surface of pristine CNT with DFT-D3. Atom color code: red: oxygen; white: hydrogen; yellow: sulfur; brown: carbon for (**a**–**c**) and grey: carbon for (**d**).

We first investigated adsorption of H_2_S molecule onto the surface of pristine CNT in order to explore the strength of interactions between these molecules and clarify whether this substrate can effectively capture and eliminate this toxic gas. Following this aim, we selected an armchair (4, 4) CNT composing of 80 carbon atoms and terminated their edge carbons by hydrogen atoms in order to eliminate the dangling bond effect. Figure 1 illustrates the optimized structure of the employed CNT model. According to this picture, the average bond distance between the two adjacent carbon atoms is about 1.44 Å, which is consistent with the experimentally measured bond distance of the CNT and the results of other theoretical works^61,62^. To explore the most stable adsorption configuration of the H_2_S molecule above the surface of CNT, two initial configurations were constructed and subjected to full structural optimization procedure in order to evaluate their relative stability by calculating the adsorption energies. Considering the fact that the H_2_S molecule has two active sites that might potentially interact with the π-extended surface of the CNT, one can conclude that the only possible orientation of the H_2_S would be the case that hydrogen atoms of the molecule approach the surface of the CNT. This is mainly caused by the fact that the H_2_S molecule is a polar gas and their hydrogen atoms are partially positively charged due to the electronegative nature of the S atom. Hence, this positively charged end of the molecule is more prone to establish electrostatic attraction with the π electrons of the CNT in comparison to the negatively charged S atom. Consequently, initial configurations were constructed in a way that molecular axis of the H_2_S molecule, which we defined here as an imaginary axis connecting two adjacent hydrogen atoms, is parallel or perpendicular with respect to the nanotube axis. Figure 1 depicts schematic representation of the constructed configuration. After performing a full structural optimization for the considered configurations, it was found that the H_2_S molecule prefers interacting with the CNT surface in parallel orientation by releasing the energy of about −0.17 eV (−3.92 kcal/mol), which is considerably weak and can be regarded as a weak physisorption. According to the optimized structure of the most stable complex as shown in Fig. 1d, the H_2_S molecule oriented above the surface of CNT in a way that its hydrogen atoms approach to the neighboring carbon atoms of the CNT with the average distance of about 2.7 Å, which is within the range of bonding distances in non**-**covalent interactions^63^. This can be further improved by considering the bond lengths of the H_2_S molecule, which remained almost fixed with respect to the corresponding values for the isolated H_2_S. To provide a clear picture of the nature of the interactions between the involved molecules, we explored the impact of dispersion forces on the overall interaction between the H_2_S and CNT. It was shown that these forces are extremely important in providing accurate geometries and adsorption energies especially in non**-**covalent interactions involving π**-**extended surfaces, which interact with various molecules. For this end, we selected the starting configuration that resulted in the energetically most stable complex. Then, we carried out a full structural optimization but without inclusion of dispersion interaction and evaluated the strength of the interaction between the involved molecules by calculating the adsorption energy and bonding distance. Figure 1e shows the optimized structure of the complex in the absence of dispersion forces and highlights the fact that the H_2_S molecule has been located far from the surface of CNT in comparison to the previously obtained distance. In addition, the adsorption energy of the H_2_S molecule was calculated to be about −0.01 eV (−0.231 kcal/mol), which is considerably different from the one obtained from the inclusion of dispersion interactions and corroborates the importance of these forces for achieving reliable geometries and adsorption energies. Hence, we could conclude that the interaction between H_2_S and CNT is of physical nature, which is totally governed by long range dispersion forces. Similar results were also reported in various theoretical works involving pristine CNT and different toxic molecules^64,65^. More detailed information about the interaction nature can be obtained by calculating the transferred charge between the involved molecules after the adsorption process. Therefore, the Hirshfeld analysis was carried out, which has been reported in several theoretical works to be a reliable choice and provide more accurate results in comparison to the other available methods^66^. With regard to the results of Hirshfeld analysis, it was found that upon the attachment of H_2_S onto the surface of pristine CNT, small charge of about 0.03 *e* was transferred between the involved fragments. This negligible charge transfer is within the domain of weak vdW governed interactions and signifies the existence of weak physical interactions between H_2_S and CNT. This is totally in line with the results of total electron density map of the complex system as shown in Fig. 2a, indicating that uniform distribution of the electron density did not alter upon the interaction between the involved molecules and there is no evidence of electron overlap in the region of H_2_S adsorption.

Moreover, electronic properties of the complex system were taken into consideration by visualizing the electron densities, which corresponded to the highest occupied molecular orbital (HOMO) and lowest unoccupied molecular orbital (LUMO), and calculating the energy gaps. It has been well understood that the conventional DFT cannot accurately describe the energy gaps; however, as demonstrated by the results of numerous theoretical works, the calculated values serve as a very helpful estimate to describe the overall trend of the system and provide accurate comparative investigations for various molecular complexes. Review of the literature showed that the (4, 4) CNT is metallic with zero energy gap; however, as the incorporated model in this work is a capped CNT, which is not periodic, the energy gap was calculated to be about 0.40 eV, which shows a semiconductor. After attaching the H_2_S molecule, the energy gap was calculated to be about 0.39 eV, which is almost the same as that for CNT, and shows that the substrate retained its inherent electronic properties after the adsorption process. Furthermore, visualization of the HOMO and LUMO electron densities, which is depicted in Fig. 2(b,c), shows that both the HOMO and the LUMO electron densities are totally distributed over the surface of CNT, while the H_2_S molecule has no contribution to the mentioned orbitals. This further represents the fact that the pristine CNT has almost a complete contribution to define overall electronic properties of the complex system, and attachment of H_2_S molecule has not any notable influence on modifying the CNT nature. These results are in line with the results reported by other theoretical works, which described changes in the electronic properties of π**-**extended surfaces upon the attachment of gases and biological molecules^67–69^.

We now explore the interaction between CH_4_ and pristine CNT as competitor of H_2_S in gasses mixture. Similar modeling and optimization procedures have been performed for adsorbed CH_4_ molecule onto the CNT surface. Our DFT**-**D3 results showed that methane molecule was attached to the pristine CNT surface above the C‒C bond perpendicular to the tube axis, with adsorption energy and equilibrium distance of −0.10 eV (−2.31 kcal/mol) and 3.07 Å (see Fig. 2d), respectively. These adsorption parameters demonstrate a non**-**covalent bonding nature, which is typical for weak physisorption. Furthermore, the adsorption of pristine CNT in H_2_S capturing is found to be higher than that of CH_4_ one, though the adsorption strength is weak.

### Interaction of H2S with functionalized CNT

**Figure 3:**
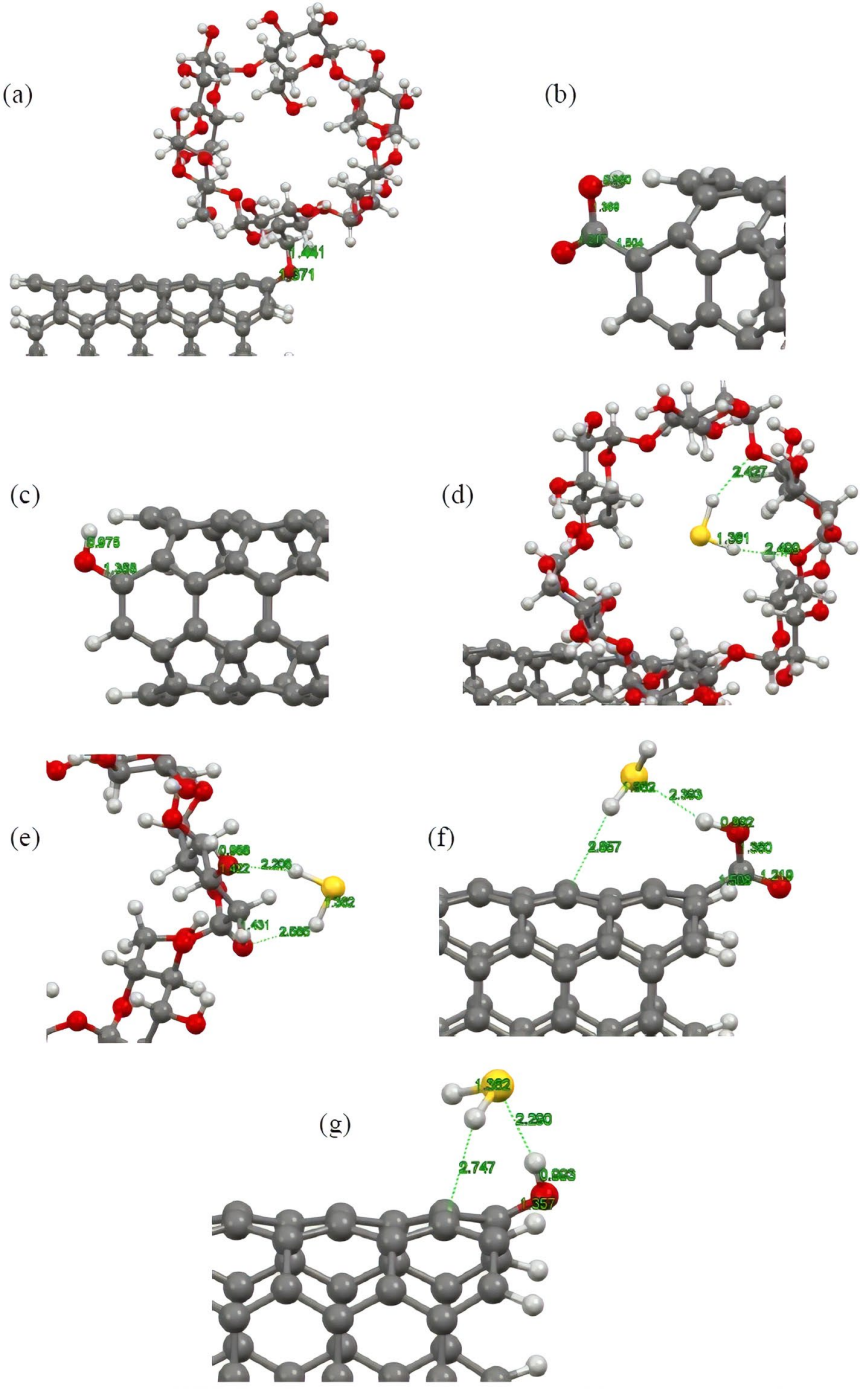
(**a**–**c**) Optimized structures of the cyclodextrin, carboxyl and hydroxyl functionalized CNTs, respectively. (**d**–**g**) Optimized structures of the H_2_S molecule interacting with cyclodextrin ((**d**) into the cavity and (**e**) onto the outer side wall), carboxyl and hydroxyl functionalized CNTs, respectively. Atom color code: grey: carbon; red: oxygen; white: hydrogen; yellow: sulfur.

As mentioned in previous section, since the adsorption strength is not sufficiently high to bind H_2_S to the surface of the carbonic substrate, the pristine CNT cannot effectively capture and eliminate the H_2_S molecule. Therefore, other techniques should be considered to enhance adsorption of the molecule while taking advantage of the excellent physical and chemical properties of the CNT. In this section, we aim at delivering a detailed analysis about the capability of the f-CNTs to capture the H_2_S molecule and investigate how and to what extent the addition of various functional groups on the terminal edges of the CNT may enhance capability of this chemically modified substrate for eliminating toxic gases. Thus, we selected three different chemical moieties; that is, hydroxyl, carboxyl, and alpha**-**cyclodextrin (CD) functional groups, and attached them to the terminal edges of the CNT by removing one hydrogen atom from the tube rim. Figure 3 depicts the optimized structures of these edge**-**f-CNTs. With regard to these optimized structures in all cases, lengths of the formed bonds between the involved entities are within the range of the covalent bond as the bond lengths are less than the sum of the covalent radii of the involved atoms. This confirms the fact that the introduced functional groups were perfectly stabilized on the CNT. After the attachment of the mentioned functional groups to the tube rim, interaction of H_2_S and these f-CNTs was investigated. Considering the existence of polar bonds and electronegative atoms within the structure of the considered functional groups, as well as the polar nature of the adsorbate, attachment of the mentioned functional groups would alter the CNT electron distribution and introduced some polar nature to the functionalized tube. This alteration in turn should change interactions between the H_2_S molecule and the CNT. To assess the extent of this change and gain a better understanding about the type of the interactions, several starting configurations were constructed for the H_2_S molecule approaching the functionalized side of the CNT in a way that the positive ends of the H_2_S molecule approached the negative ends of the functional groups and vice versa. For the cyclodextrin functional group, the H_2_S molecule was initially placed both inside and outside the ring in various possible orientations based on the mentioned procedure. The rationale behind using the mentioned approach regarding the construction of initial configurations lies in the fact that the attached chemical groups would provide the capability to the CNT to interact with the guest molecules through electrostatic attractions in addition to the dispersion interactions. Consequently, among all of the possible orientations, only those ones for the H_2_S molecule were considered, which were more prone to establish electrostatic attraction with the f-CNT and excluded the rest from further investigations for the sake of computational costs. After constructing the starting configuration, we carried out a full structural optimization similar to the previous section for the complex systems and evaluated their stability by calculating the adsorption energies and equilibrium distances. Figure 3(d–g) shows the optimized structures of the energetically most stable complexes. After a full structural optimization, it was found that the H_2_S molecule was adsorbed onto the f-CNTs upon the functional groups with the adsorption energy of about −0.363 and −0.486 eV for the hydroxyl (OH**-**CNT), and carboxyl (COOH**-**CNT) systems, respectively. In the case of cyclodextrin functionalized CNT (CD**-**CNT), the calculated adsorption energy was determined to be about −0.544 and −0.394 eV for the adsorbed H_2_S molecule into/onto the cavity/outer site of cyclodextrin entity. It can be seen in all the cases that the amount of the calculated adsorption energies is about two times higher than that of the one obtained for pristine CNT, and shows that stronger interaction took place between the involved molecules. However, despite the observed enhancements, the magnitude of the adsorption energies is still within the range of physisorption but this time it is considerably stronger than the previously obtained value. This enhancement in the adsorption energy can effectively improve the H_2_S adsorption capability of the CNT and also restrict movement of the adsorbed H_2_S above the surface of substrate, which serve as a crucial criterion for an effective and applicable adsorbent. Moreover, the magnitude of the adsorption energies is in the way that it can easily assist desorption of adsorbate, which asserts feasibility of the f-CNTs as proper candidates for long term usage. Taking a closer look at the optimized structures reveals important facts about the magnitude of the adsorption energies. This, to some degree, justifies lower level of adsorption energy of H_2_S onto the OH-CNT and COOH-CNT in comparison to that into the cavity of cyclodextrin for the CD**-**CNT system. For the hydroxyl and carboxyl ones, the optimized structures of the complex system are such that the H_2_S molecule not only approaches the functionalized ends of the CNTs through hydrogen bonding, but there is also some dispersion as well as electrostatic forces involved in the total interaction. The same result can also be applied for the optimized structure of the H_2_S molecule within the ring of cyclodextrin group as the H_2_S molecule is surrounded by a large number of atoms in its vicinity, which increases dispersion interactions magnitude and hence enhances total adsorption energy. These results can be quantitatively seen by calculating the contribution of dispersion interactions to the considered complexes where the adsorption energies in the absence of dispersion forces were calculated to be about −0.10 and −0.02 eV for the COOH-CNT and OH-CNT, respectively, while this value dropped to +0.05 eV for the incorporated H_2_S molecule into the cyclodextrin in CD**-**CNT. The calculated values reveal the importance of dispersion forces together with the electrostatic interactions in stabilizing the adsorbate molecule on the f-CNTs and clearly illustrate that the magnitude of the dispersion interactions is directly proportional to the size, mass, and surface area between the interacting molecules.

**Figure 4:**
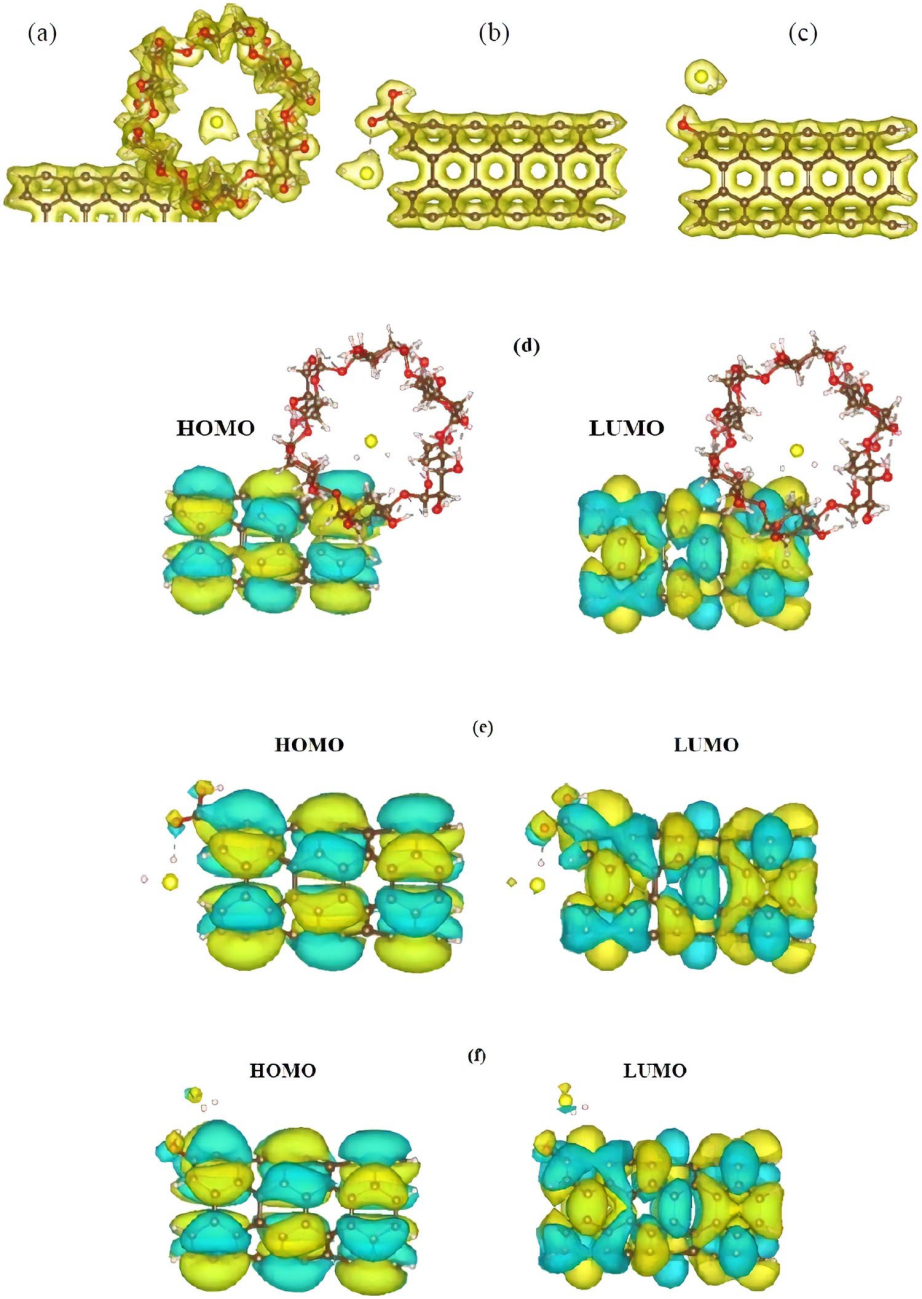
Total electron density maps of the (**a**) cyclodextrin (**b**) carboxyl and (**c**) hydroxyl functionalized CNTs interacting with the H_2_S molecule in energetically favourable states. (**d**–**f**), respectively, corresponding HOMO and LUMOs of the cyclodextrin, carboxyl and hydroxyl functionalized CNTs interacting with the H_2_S molecule. Atom color code: red: oxygen; white: hydrogen; yellow: sulfur; brown: carbon.

To gain a deeper insight of the interactions nature, we plotted the total electron density maps of the considered complexes (Fig. 4). According to the figure, the electron cloud for all the complexes is uniformly distributed over the individual molecules and there is no evidence in the electron overlap between the contributing entities. This finding is consistent with the results of charge transfer analysis, which informs the transfer of 0.05 to 0.07 *e* from the H_2_S to the f-CNTs. Isosurface plots of HOMO and LUMO electron densities were calculated and represented in Fig. 4(d–f). Similar to the situation for pristine CNT, the majority of both the HOMO and the LUMO orbitals were distributed over the f-CNTs, and the H_2_S molecule had no contribution to the HOMO and LUMO orbitals of the complex systems. This also resembles the existence of physical interactions between the considered molecules since we could not find any overlaps between the corresponding orbitals of the constituting fragments in the complex system that is in line with the results reported by various theoretical works as well as the magnitude of the calculated adsorption energies and transferred charges. Electronic properties of the systems were also taken into consideration in terms of HOMO**-**LUMO energy gap. The results suggested that intrinsic electronic properties of the f-CNTs remained unaltered after adsorption of H_2_S molecule. Indeed, for all the considered functionalized CNTs, the calculated HOMO**-**LUMO gap ranged between 0.38 eV to 0.4 eV, which indicates that the CNTs retained their original semi**-**conducting nature upon the attachment of functional groups as well as the H_2_S molecule, and functionalization process could not introduce any improvement to the pristine CNT to be potentially useful in sensing applications.

**Figure 5:**
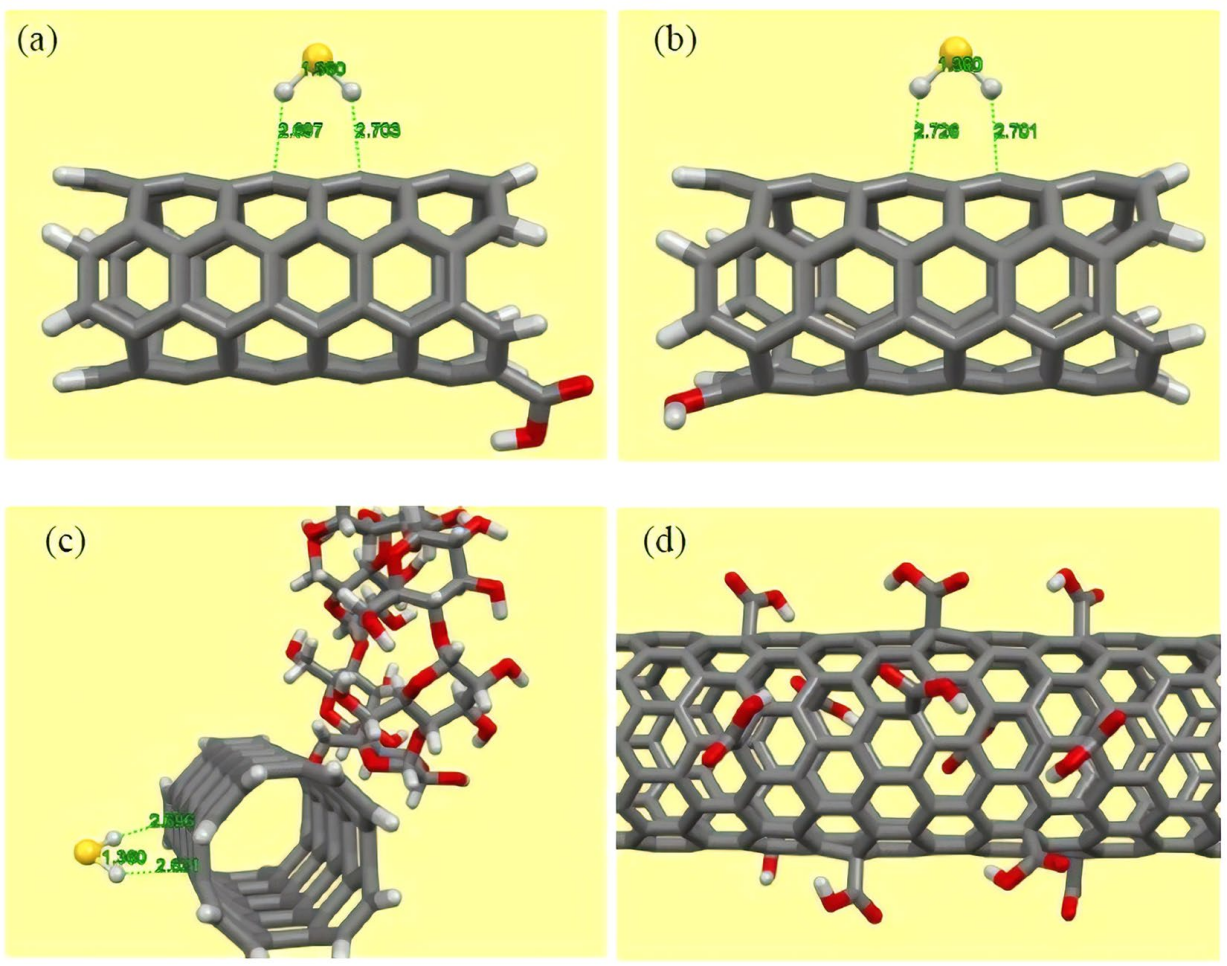
Optimized structures of the H_2_S molecule attached to tube surface of (**a**) COOH-CNT, (**b**) OH-CNT, and (**c**) CD-CNT. (**d**) Conceptual model for functionalized CNT with carboxyl groups on the tube surface. Atom color code: grey: carbon; red: oxygen; white: hydrogen; yellow: sulfur.

For comparison, we have also evaluated the adsorption ability of tube surface of f-CNTs in H_2_S adsorption. After full structural optimization of modeled systems, the calculated adsorption energies revealed that H_2_S bounds weakly to the surface of f**-**CNTs while the adsorption strength is as magnitude as of the pristine CNT (−0.17, −0.16 and −0.17 eV for OH**-**CNT, COOH**-**CNT and CD**-**CNT systems, respectively). Figure 5 demonstrates the optimized structures of adsorbed H_2_S onto the nanotube surface of f**-**CNT systems with DFT**-**D3 level of theory. These results indicated that functional groups have significant role in the H_2_S adsorption and their coverage on the CNTs can affect the adsorption capacity of considered adsorbents. It should be noted that CNTs may be functionalized by several groups at the open/end of nanotube (the present work) as well onto the tube surface as demonstrated in Fig. 5d (suggested study for the future works). A less adsorption energy of COOH**-**CNT system may be attributed to the electron affinity of carboxyl group and hence, inferior electron accommodation on the CNT surface compared to the pristine CNT and OH**-**CNT systems.

### Interaction of CH4 with functionalized CNT

**Figure 6:**
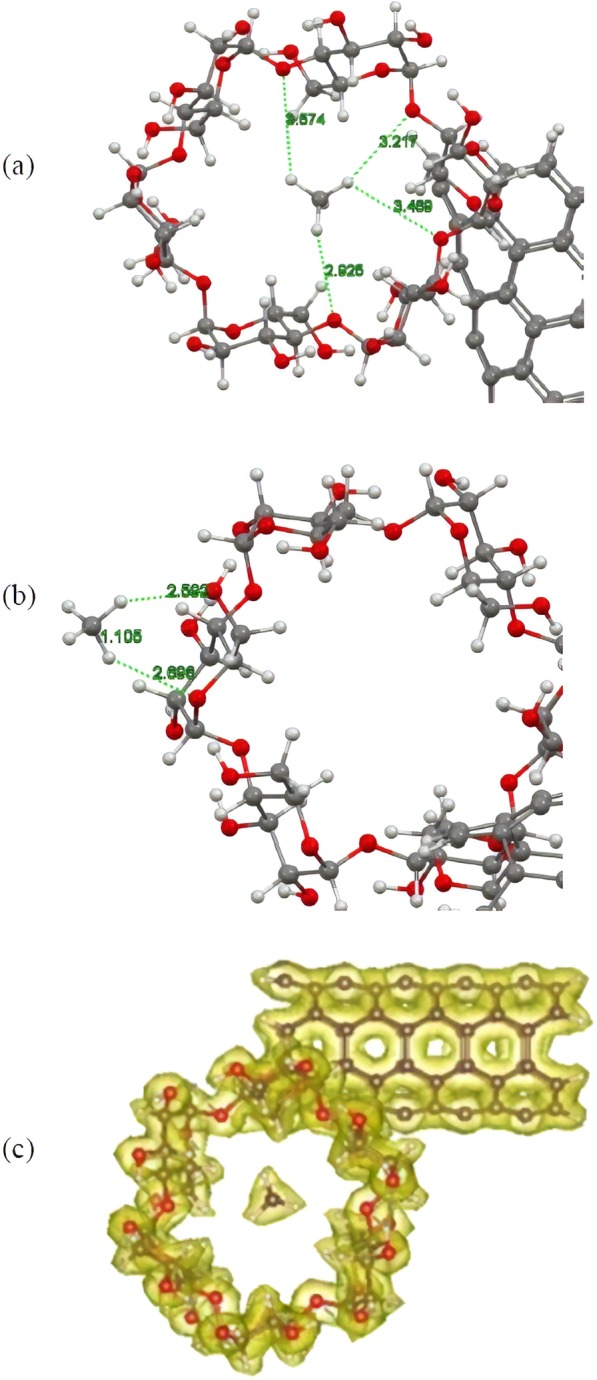
Optimized structures of the CH_4_ molecule interacting with (**a**) inner site and (**b**) outer side wall of cyclodextrin functionalized CNT. (**c**) Total electron density map of the CH_4_ incorporated into the CD**-**CNT. Atom color code: red: oxygen; white: hydrogen; yellow: sulfur; grey: carbon for (**a**), (**b**) and brown: carbon for (**c**).

By considering the CD**-**CNT as the most effective substrate for eliminating H_2_S molecule either for adsorption strength or several potential active sites, we planned to study the comparative adsorption of CH_4_ molecule with respect to the H_2_S using this substrate in order to simply check whether the CD**-**CNT can selectively separate a mixture of CH_4_ and H_2_S gases and also explore the extent and nature of the interactions between CH_4_ and this substrate. Then, we constructed several initial configurations for the methane molecule approaching various potential adsorption sites of the cyclodextrin and CNT, including inside and outside the cyclodextrin ring and surface of the CNT in various positions. Afterwards, a complete structural optimization was carried out for the considered adsorption configurations. The relative energetic stabilities of the considered structures were evaluated by the adsorption energy calculations, and the most stable adsorption pattern was found to be the methane molecule placed inside the cyclodextrin ring (Fig. 6a). The BSSE**-**corrected adsorption energy was calculated to be about −0.14 eV, which is about 2.5 times smaller than that of the most stable pattern of H_2_S, and shows that the methane adsorption strength is much weaker than the adsorption strength of H_2_S molecule. Meanwhile, the energetically favorable state for the CH_4_ molecule was adsorbed onto the outer site of the cyclodextrin (Fig. 6b) accompanied with release of about −0.076 eV energy that indicates a poor adsorption ability of the outer sidewall of CD**-**CNT in methane capture. Comparison of the adsorption energies revealed that this functionalized substrate exhibits different affinity toward different gas molecules, which mainly stems from the differences in chemical properties of the interacting gases. Being a highly polar molecule, adsorption of H_2_S gas is mainly dominated by electrostatic interactions as the consequence of the existence of polar bonds and electronegative centers within the structure of cyclodextrin; however, the CH_4_ molecule does not show any polar nature, and interactions between methane and cyclodextrin is almost purely dispersion**-**governed. This is partially evident from the average distance of CH_4_ with respect to the inner/outer site of cyclodextrin after the adsorption process, which is 2.65/2.64 Å and is typical for a weak physisorption.

The results obtained from Hirshfeld analysis can also corroborate the existence of weak interactions between the involved molecules by suggesting the transfer of about 0.03 *e* between methane and the f-CNT. This result can be further clarified by calculating the electron density distribution of the complex system as shown in Fig. 6c. Uniform distribution of the electron clouds is observed after adsorbing methane molecule. In addition, there is no overlap between the electron densities anywhere near or exactly at the adsorption site. To provide a clear**-**cut illustration about the role of dispersion interactions on the overall adsorption behavior of methane molecule into this substrate, we re**-**optimized that configuration corresponding to the energetically most stable adsorption without inclusion of dispersion interactions. With regard to the optimized structure, equilibrium geometry of the adsorbed methane molecule (both in equilibrium distance and final orientation of the adsorbed methane molecule) strongly deviates from the geometry that the dispersion interactions have been considered. On the other hand, adsorption strength was strongly affected by eliminating dispersion interactions and the energy was calculated to be about + 0.02 eV, which is considerably smaller (weaker) than that of the dispersion**-**corrected energy (−0.14 eV). Altogether, the above results highlight the dominating role of dispersion forces in the adsorption of methane molecule in contrast to the H_2_S adsorption where the electrostatic forces were the main contributors.

Adsorption of CH_4_ molecule by OH**-**CNT and COOH**-**CNT systems showed that methane interacts weakly with both hydroxyl and carboxyl active sites with adsorption energies of about −0.11 and −0.09 eV, respectively. The bonding distances between nearest atoms, the H atoms of CH_4_ and the O/C atom of f**-**CNTs, have been estimated to be about 2.9 Å for both systems under study. Further evaluating about the interaction of methane with the nanotube surface of f**-**CNTs indicates similar weakness strength for f**-**CNT in CH_4_ adsorption either, onto the functional groups or the nanotube middle surface. Calculated adsorption energies of optimized configurations are determined to be about −0.10 eV for methane adsorbed onto the tube surface of both OH**-**CNT and COOH**-**CNT systems indicating that functional groups could not affect the CH_4_ adsorption of selected substrates.

### Molecular dynamics simulation of H2S-CH4/CD-CNT system

**Figure 7:**
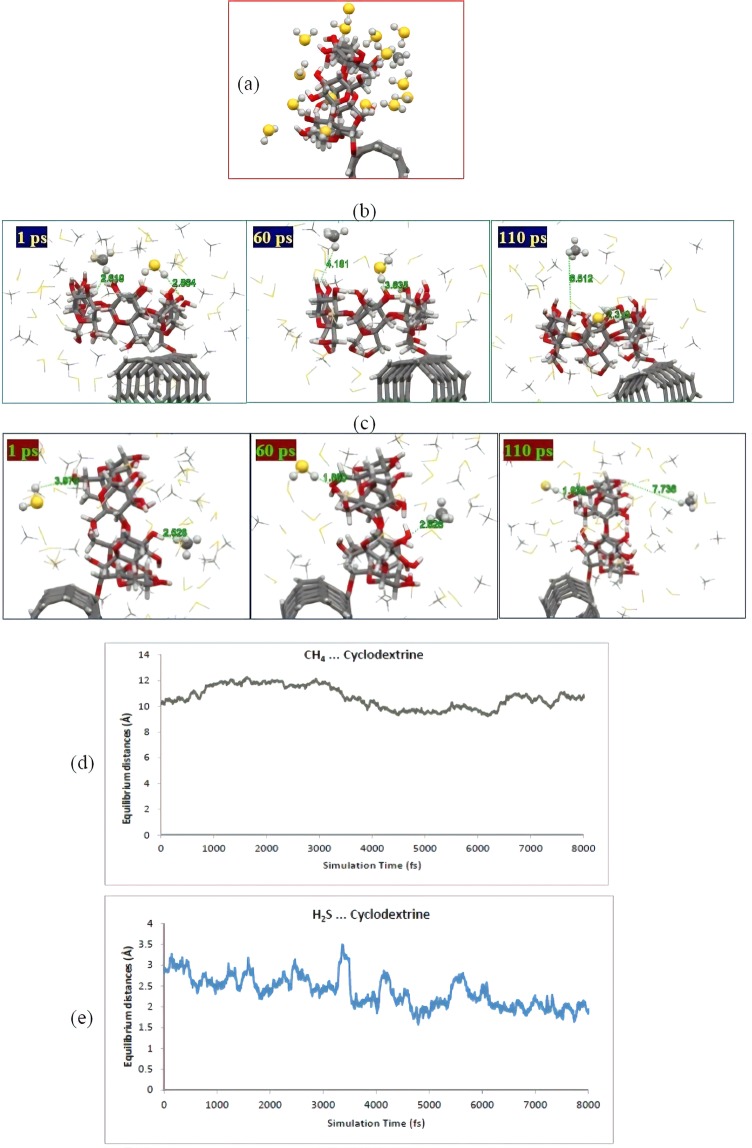
Schematic representation of snapshot of (**a**) the cyclodextrin-functionalized CNT consisting of 30H_2_S/30CH_4_ molecules at 150 ps of simulation, (**b**) a H_2_S/CH_4_ molecule adjacent to the cavity of cyclodextrin and (**c**) a H_2_S/CH_4_ molecule near the side wall of cyclodextrin during the specified simulation time steps. (**d**) Equilibrium distance between S atom from H_2_S and nearest H atom of the CD and (**e**) Equilibrium distance between H atom from CH_4_ and nearest O atom of the CD moiety during the 8 ps of the tail**-**end of simulation times. Atom color code: grey: carbon; red: oxygen; white: hydrogen; yellow: sulfur.

In this section, molecular dynamics (MD) was simulated to evaluate the CD**-**CNT system capability for efficient adsorption of H_2_S molecules in H_2_S/CH_4_ mixture. In order to achieve this important aim, we used DFTB based MD simulation for CD**-**CNT system surrounded by 30 H_2_S and 30 CH_4_ molecules as shown in Fig. 7a. We optimized the modeled system at temperature 300 K to simulate the ambient condition for system under study.

Our quantum mechanics-based simulation after 150 ps of simulation time steps showed that up to 14 H_2_S molecules could be attached to the cyclodextrin moiety, while only one CH_4_ molecule bonded to cyclodextrin. The equilibrium distances were considered to be about 2.5 Å or less for the neighbor molecules to cyclodextrin. It should be mentioned that higher levels of H_2_S molecules could be attached to the adsorbent, and H_2_S molecules near the cyclodextrin (bilayers adsorption) were not accounted here. It was found that two of the adsorbed H_2_S molecules were incorporated into the cyclodextrin cavity during the simulation process. Meanwhile, methane molecules are spread out and placed away from the cyclodextrin entity. In addition, we accounted for the adsorbed molecules onto the cyclodextrin at about 200 ps and found that the number of H_2_S and CH_4_ molecules is the same as the previously accounted ones at 150 ps that indicates a stable state for system under simulation.

For further clarification, we have demonstrated both initial and final configurations of an attached H_2_S/CH_4_ molecule as well as one H_2_S molecule encapsulated into the cyclodextrin in Fig. 7b,c. According to Fig. 7b, after 110 ps of simulation time, the H_2_S molecule placed at about 2.8 Å moved toward the cyclodextrin cavity, while the situation of CH_4_ molecule with a similar situation getting away the cyclodextrin changed from 2.6 to 6.5 Å during the optimization procedure. We observed a similar trend for some H_2_S and CH_4_ molecules located around the cyclodextrin as depicted in Fig. 7c, where H_2_S was housed near the cyclodextrine during the simulation procedure, while CH_4_ molecule left the cyclodextrine skeleton domain and placed about 7.7 Å apart from the adsorbent. Meanwhile, intermolecular distances between adjacent H_2_S/CH_4_ molecule and cyclodextrin sidewall were calculated for eight ps of the tail-end of simulation steps and represented in the figure. The obtained graphs reveal a relative stable state for the attached H_2_S molecule as well as the receded CH_4_ molecule during the simulation times. Equilibrium distance for the adsorbed H_2_S molecule (distance between S atom from H_2_S and the nearest H atom of the CD) was estimated to be about 2.5 Å, while the corresponding value for CH_4_ molecule (distance between H atom from CH_4_ and the nearest O atom of the CD) is around 11 Å. These findings demonstrated that the functionalized CNT with cyclodextrin can be a superior nanomaterial for H_2_S adsorption and removal from the H_2_S/CH_4_ for industrial applications. Therefore, we suggested practical evaluation and possible application of this novel nanostructured material by convenient available techniques.

## Conclusion

With the aim of developing a novel hybrid material to selectively eliminate toxic gases from a mixture of different gas molecules, the selective adsorption of H_2_S molecule, as one of the most hazardous toxic gases, was investigated in light of DFT calculations. For this end, pristine and chemically modified CNTs were considered as candidates to fully understand the effect of covalent functionalization on the adsorption nature and affinity of the CNT toward various gas molecules. The adsorption strength of the H_2_S gas on the functional groups as well as the middle of nanotube was estimated by calculating the adsorption energy and compared to that of the methane molecule. In all the cases, functionalization of the CNT significantly improved the adsorption capability of the CNT by introducing additional bonding sites with specific chemical affinity toward different gas molecules. Since the H_2_S molecule is a highly polar gas, attachment of functional groups containing electronegative atoms can significantly enhance the affinity of H_2_S toward the CNT rather than the CH_4_ molecule. This difference in affinity has been raised as the adsorption energy difference of the respective molecules onto the CNT and can be potentially useful in developing efficient hybrid adsorbents for the selective separation and elimination of toxic gases. MD simulation at the DFTB level of theory was carried out under ambient conditions for CD**-**CNT consisting of H_2_S/CH_4_ mixture. It was found that CD**-**CNT is exhibited as a superior material in H_2_S adsorption where up to 14 H_2_S molecules could be adsorbed by cyclodextrin moiety, while only one CH_4_ molecule, as its competing entity, is attached to the cyclodextrin under ambient conditions.
